# Alcohol Use Disorder Following Bariatric Surgery: A Narrative Review

**DOI:** 10.62641/aep.v53i3.1830

**Published:** 2025-05-05

**Authors:** Farhana Nazmin, Sasidhar Gunturu, Sanobar Jaka, Saidur Rahman

**Affiliations:** ^1^Department of Psychiatry, Bronx Care Health System, New York, NY 10457, USA; ^2^Department of Psychiatry, Icahn School of Medicine at Mount Sinai, New York, NY 10029, USA; ^3^Department of Psychiatry and Behavioral Sciences, Nassau University Medical Center, East Meadow, NY 11554, USA; ^4^Department of Population Health, NYU School of Medicine, New York, NY 10016, USA; ^5^Middle Tennessee Mental Health Institute, Nashville, TN 37214, USA

**Keywords:** alcoholism, bariatric surgery, gastrectomy, gastric bypass, nutritional status

## Abstract

**Objective:**

To explore the incidence of alcohol use disorder (AUD) after bariatric surgery and the associated risk factors and specific manifestations of increased AUD risk, such as increased alcohol sensitivity and earlier symptom onset.

**Methods:**

This review adhered to PRISMA guidelines. Data were sourced from PubMed, Google Scholar, Cochrane, and Science.gov using search terms related to alcohol, bariatric surgery, and nutrition. Articles were screened based on inclusion and exclusion criteria: Inclusion criteria were English language articles published from 2013–present, availability in full text or open access, and adult patients (≥18 years) who had bariatric surgery. Exclusion criteria were non-English articles, articles written before 2013, unavailable full text or open access, articles that featured pediatric patients or patients who did not use alcohol or had bariatric surgery, and abstracts or opinion pieces. A final total of 11 studies were found. Data Extraction: Studies published from 2013 to the present that involved adult bariatric surgery patients with a history of alcohol consumption.

**Results:**

Findings suggest an elevated risk of AUD post-bariatric surgery, particularly following sleeve gastrectomy or Roux-en-Y gastric bypass surgery. There was strong evidence of increased alcohol sensitivity and AUD symptoms post-surgery, causing potential health effects.

**Conclusions:**

These results underscore the importance of comprehensive preoperative assessments and tailored interventions for bariatric surgery patients with AUD.

## Introduction

Severe obesity is a serious health condition defined by a body mass index (BMI) >30. In the United States, the prevalence of obesity among adults in the United States rapidly increased between 1980 and 2000 [1]. Currently, ~35% of adults in the United States have obesity [2]. Weight loss is related to short-term improvement and prevention of metabolic and cardiovascular-related issues [1]. Additionally, techniques that are less invasive than the Roux-en-Y gastric bypass (RYGB) have been introduced with research showing short-term improvement, such as adjustable gastric band (AGB) and sleeve gastrectomy (SG) [3]. Long-term outcomes of these interventions are available from observational studies and recent randomized controlled trials (RCT) that directly compared bariatric procedures to medical and lifestyle interventions for patients with type 2 diabetes (T2DM) [3, 4, 5]. However, even though evidence confirms short-term effectiveness and safety of bariatric surgery for weight loss and T2DM remission, there are uncertainties about long-term complications that are not fully understood.

Several long-term complications following bariatric surgery have been identified in the literature, in addition to the immediate risk of developing alcohol use disorder (AUD). One of the most concerning complications is liver disease, such as alcoholic hepatitis or cirrhosis, which can result from chronic alcohol consumption, particularly in patients whose ability to metabolize alcohol is altered post-surgery. Another significant long-term complication is nutritional deficiencies—bariatric surgery already places patients at risk for malabsorption of key nutrients, and alcohol use can exacerbate this issue. Chronic alcohol consumption can interfere with the absorption of essential vitamins and minerals like vitamin B12, calcium, and iron, which can lead to vitamin deficiencies and related conditions, such as anemia, osteoporosis, and neurological disorders. Moreover, mental health issues can arise as a long-term complication. Patients may experience heightened rates of depression, anxiety, and social isolation, which can, in turn, hinder their ability to maintain healthy habits and exacerbate their alcohol consumption. Finally, weight regain is a critical concern since alcohol is calorie-dense and offers no nutritional value and could undermine the initial success of bariatric surgery. Weight regain not only affects the physical outcomes of the surgery but can also have psychological impacts, including frustration and a potential relapse into unhealthy behaviors. These long-term complications underscore the importance of continuous monitoring and comprehensive care for patients who undergo bariatric surgery to mitigate the risks associated with AUD.

Overall, bariatric surgery can induce drastic gut changes due to subsequent weight loss that may influence how the body responds to food and absorbs nutrients. Poor absorption will lead to longstanding nutritional deficiencies that require lifelong nutritional surveillance and supplementation. Bariatric surgery can also change the way the body processes and absorbs certain substances, such as alcohol—gut physiology changes can increase the speed of absorption and sensitivity to alcohol, which is especially problematic for people at risk for alcohol use disorder (AUD).

Since bariatric surgery modifies the way nutrients are metabolized and absorbed, preexisting nutritional deficiencies typical of people with AUD could be potentially aggravated. Furthermore, the quick absorption of alcohol after surgery may increase the risk of complications of alcohol consumption and disrupt the absorption of nutrients. It is therefore critical to understand how AUD and bariatric surgery interact to optimize postoperative care plans and reduce unfavorable outcomes in this population.

Because altered gastrointestinal physiology, nutritional deficiencies, and alcohol metabolism may intersect, this narrative review aimed to examine the available literature for studies that examined patients with AUD after bariatric surgery. From these studies, we sought to understand the post-bariatric surgery incidence of AUD and to identify risk factors associated with poor nutritional status post-surgery.

## Methods

We consulted the Preferred Reporting Items for Systematic Reviews and Meta-analyses (PRISMA) guidelines for the methodology of this narrative review [6]. Inclusion criteria for the study were English language human studies, including systematic reviews, case reports, and observational studies, published between 2013 (10 years prior to when this study was conducted) to the present. Patient samples in these studies were adults (≥18 years) and had prior bariatric surgery and AUD. The final inclusion criterion was full-text or opened access journal articles available to the authors. Exclusion criteria were abstracts, textbook chapters, opinion pieces, articles not written in English, articles published before 2013, and animal studies. We also excluded studies that featured pediatric patients, patients who did not use alcohol, and patients who did not have bariatric surgery. The databases searched included PubMed, Google Scholar, Cochrane Reviews, and Science.gov. Searches were conducted between January 4 and January 30, 2024.

The search strategy included keyword and Medical Subject Headings (MeSH) searches in PubMed and keyword searches for the other three databases. Search strings for each of the four databases are found in Table 1. The method of selecting articles consisted of automatic removal of duplicates, automatic removal of exclusions, followed by manual review of each article by the authors to screen for inclusion or exclusion. The following data items were collected from each study: study location, type of study design used, sample size(s), type of intervention used, and overall results of the study.

**Table 1.** **Database search strategies**.

Database	Search strategy
PubMed	alcohol consumption OR alcohol drink OR (“alcohol drinking/adverse effects”[Majr] OR “alcohol drinking/metabolism”[Majr] OR “alcohol drinking/pathology”[Majr] OR “alcohol drinking/physiopathology”[Majr]) AND (nutritional profile OR metabolic profile OR alcohol physiology OR alcohol metabolism OR “nutritional status/physiology”[Majr]) AND “bariatric surgery/education”[Majr]
Cochrane	alcohol consumption OR alcohol use OR alcohol drink AND nutritional profile OR metabolic profile OR alcohol physiology OR alcohol metabolism AND bariatric surgery, gastric bypass, gastric sleeve
Science.gov	alcohol use AND bariatric surgery
Google Scholar	alcohol use AND bariatric surgery AND Nutritional status

Abbreviation: Majr, major topic Medical Subject Headings (MeSH) term.

## Results

**Fig. 1.**
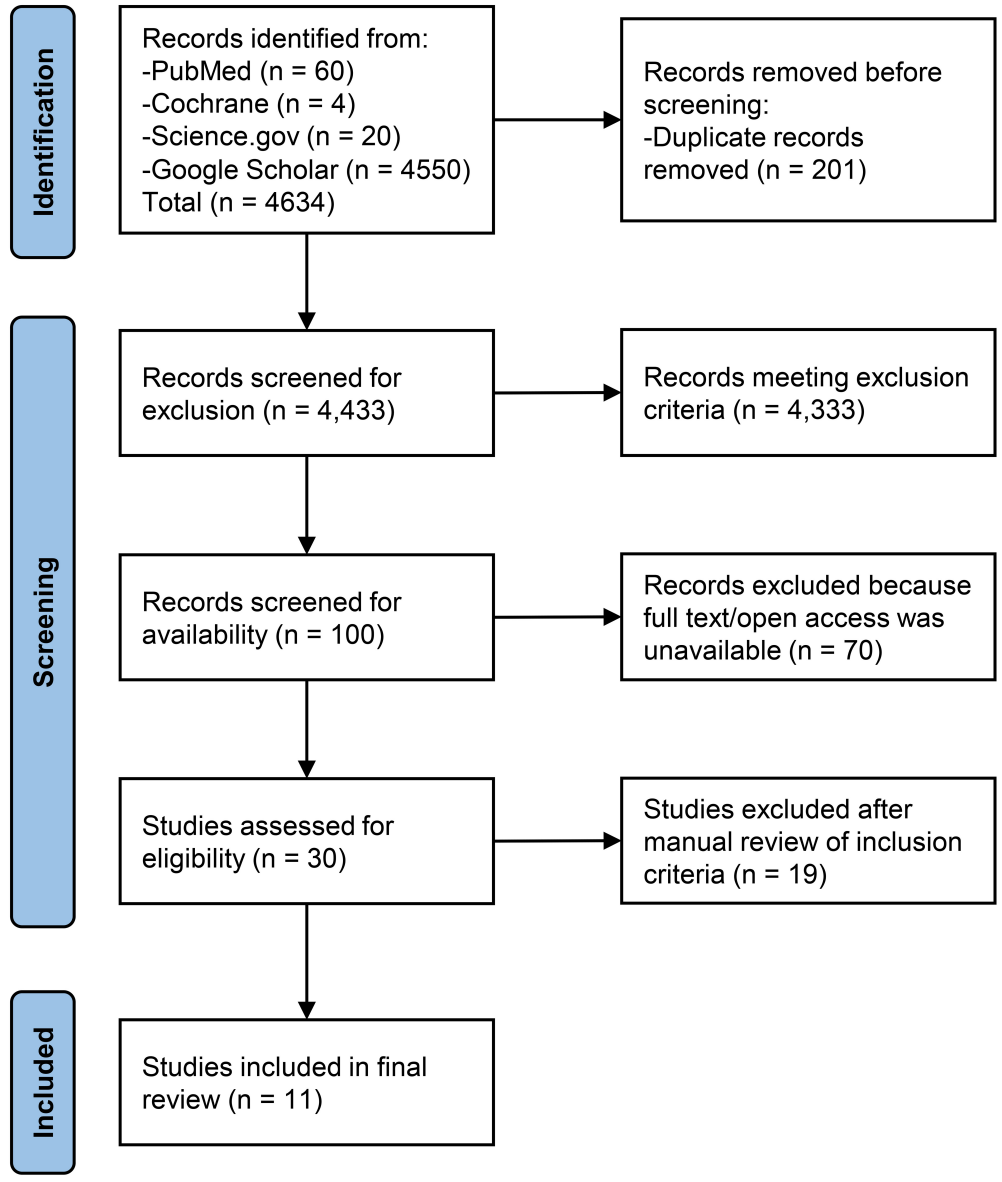
**Preferred Reporting Items for Systematic Reviews and Meta-analyses (PRISMA) flow diagram depicting exclusions of studies to reach final amount**.

Initial searches retrieved records for 60 articles from PubMed, 4 articles from Cochrane Review, 20 from Science.gov, and 4550 from Google Scholar for a total of 4634 articles (Fig. 1). After 201 duplicate records were removed, 4433 records remained for screening. Of these, 4333 were excluded because they met our exclusion criteria, leaving 100 studies. Seventy studies were excluded for not being available as full text or open access. The final 30 studies were manually reviewed for inclusion criteria by the authors, who excluded 19, leaving a final total of 11 studies. From these, we conducted our narrative review. Characteristics of the studies can be found in Table 2 (Ref. [7, 8, 9, 10, 11, 12, 13, 14, 15, 16, 17]).

**Table 2.** **Studies included in narrative review**.

Study	Location	Type of study	Sample size	Result
King *et al*., 2012 [7]	USA	Longitudinal cohort study	1945 participants	Increased alcohol sensitivity post-RYGB, combined with a return to excessive alcohol intake in the second year after surgery, contributed to the escalation of AUD.
Siikaluoma *et al*., 2022 [8]	Sweden	Retrospective cohort study	410 participants	The rate of excessive alcohol consumption at the 2-year mark after sleeve gastrectomy or RYGB was 8.3%.
Smith *et al*., 2018 [9]	United Kingdom	Cohort study (interview and self-assessment)	26 participants	Individuals had reported higher subjective sensitivity after surgery to alcohol.
Bramming *et al*., 2021 [10]	Denmark	Prospective cohort study	13,430 participants	Those who had had bariatric surgery, predominantly gastric bypass (95%), had a risk of AUD 6–7 times greater compared to non-surgical controls.
			21,021 controls	
Briegleb and Hanak, 2020 [11]	Belgium	Basic literature review of PubMed and Sciencedirect	Not mentioned	The likelihood of developing AUD increases following RYGB. Other risk factors included male sex, younger age, smoking, regular alcohol consumption, previous AUD diagnosis, recreational drug use, low sense of belonging.
Mahmud *et al*., 2023 [12]	USA	Retrospective cohort study	1854 participants	Patients who received RYGB experienced a higher likelihood of hospitalization related to AUD compared to those who had undergone sleeve gastrectomy.
Cuellar-Barboza *et al*., 2015 [13]	USA	Longitudinal cohort study (with self-assessment)	41 RYGB patients	Patients can develop progressive AUD years after RYGB. Some patients began consuming alcohol at 17 months post-surgery; they met AUD diagnostic criteria by 37 months. 4.9% of alcoholics seeking treatment had prior RYGB.
			122 controls	
Pepino *et al*., 2015 [14]	USA	Cohort study	17 total females	RYGB increased the rate of alcohol absorption. Participants had earlier and higher blood alcohol content and increased feelings of inebriation.
			5 females followed longitudinally	
Reaves *et al*., 2019 [15]	USA	Qualitative interviews with qualitative thematic analysis	14 total participants	Post-surgical themes contributing to problematic alcohol consumption: coping and disinhibition, negative self-image, restrictions on eating behavior, felt unprepared for the effects of surgery, not seeking emotional support for problematic alcohol consumption, instrumental support from medical team ending after 2 years.
			9 females	
			5 males	
				Nonproblematic post-surgical themes: social drinking and maintaining control, positive self-image, felt prepared for the effects of surgery in hindsight, seeking support emotional, receiving instrumental support from close people, self-confidence (resilience).
Tvedt *et al*., 2023 [16]	Norway	Qualitative interviews	10 total participants	Bariatric surgery may have increased participants’ susceptibility to problematic alcohol consumption. Post-surgery, alcohol was used as an appetite stimulant, a replacement for food, and a pain reliever.
			4 females	
			6 males	
White *et al*., 2024 [17]	USA	Commentary	350	Post RYGB and SG, which are the most performed bariatric surgeries, patients are more susceptible to postoperative alcohol-related complications compared to other bariatric surgeries and their pre-surgery status.

Abbreviations: AUD, alcohol use disorder; RYGB, Roux-en-Y gastric bypass; SG, sleeve gastrectomy.

### Alcohol Consumption After Bariatric Surgery

King *et al*. [7] examined individuals who underwent different types of bariatric surgical procedures, such as RYGB, laparoscopic adjustable gastric banding (LAGB), sleeve gastrectomy, biliopancreatic diversion with duodenal switch, or banded gastric bypass. They investigated the relationship between RYGB and AUD compared to LAGB, focusing on alcohol consumption and AUD. The study results showed a significant increase in alcohol consumption frequency during the second postoperative year among participants who underwent RYGB or LAGB, compared to the year before surgery or the first postoperative year.

Cuellar-Barboza *et al*. [13] concluded that patients develop progressive AUD several years following RYGB. Pepino *et al*. [14] found that RYGB increased the rate of delivery of alcohol, which resulted in earlier alcohol use and higher blood alcohol content (BAC). Participants self-reported that they felt stronger feelings of drunkenness compared to before surgery. Tvedt *et al*. [16] found that participants reported how their body responded to alcohol differently post-surgery. Participants experienced problematic alcohol consumption after bariatric surgery and felt intense intoxication compared to their pre-surgical state. Their participants used alcohol as an appetite stimulant, food replacement, and pain reliever [16].

### Post-Bariatric Surgery Incidence of AUD

King *et al*. [7] found no significant difference of AUD incidence in their sample before surgery and at 1 year after surgery. However, they noted a significant increase in AUD incidence 2 years after surgery. Cuellar-Barboza *et al*. [13] found that patients developed progressive AUD in the years after RYGB. Some of their patients began consuming alcohol around 17 months post-surgery. By 37 months, many patients met diagnostic criteria for AUD, which required treatment at an addiction facility around 65 months.

Smith *et al*. [9] found that all participants (100%) in their study met criteria for problematic alcohol use. Additionally, the authors found that about one-third of their participants developed new-onset AUD after surgery. Bramming *et al*. [10] reported that the greatest risk of developing AUD was more than 5 years after surgery. They found that those who had bariatric surgery had a 6–7 times higher risk of AUD compared to non-surgery controls. The authors also found an incidence rate of 3.7% at 5 years post-surgery compared with only 0.8% for individuals who did not have surgery. At 10 years, the incidence rate increased to 7.8% for those who had surgery compared with only 1.4% for those who did not.

Tvedt *et al*. [16] found that their participants experience problematic alcohol consumption following bariatric surgery. Siikaluoma *et al*. [8] studied alcohol overconsumption as measured with phosphatidylethanol 16:0/18:1 as a screening tool and found a problematic alcohol use rate of 3.0% before surgery, which increased to 8.3% 2 years after surgery. According to White *et al*. [17], individuals having bariatric procedures faced a higher risk of developing AUD post-surgery. Additionally, the findings from the Longitudinal Assessment of Bariatric Surgery-2 (LABS-2) study (which the White *et al*. [17] study used for sampling) found that the occurrence of symptoms related to AUD increased from 7% before surgery to 16% 7 years after surgery [18].

### Factors That Contribute to Post-Operative AUD

Several studies examined risk factors that were found to increase the likelihood of participants developing AUD. King *et al*. [7] found similar pre-operative risk factors, including being male, being at a younger age, regularly consuming alcohol, and using drugs recreationally. The authors also noted that having an RYGB was a post-surgical risk factor. Cuellar-Barboza *et al*. [13] reported that men developed AUD and sought treatment earlier than women. They found that 4.9% of those who sought treatment for problematic alcoholic use had an RYGB procedure.

Reaves *et al*. [15] conducted a qualitative thematic analysis for patients who experience problematic alcohol use after gastric bypass. The themes that the authors found in their patients’ responses included several factors that contributed to problematic alcohol consumption: using alcohol as a coping tool and feeling more disinhibition, having a negative self-image, using alcohol to supplement the food restricted following surgery, feeling like they were unprepared for the effects of surgery, not seeking emotional support for their problematic alcohol consumption, and medical support ending after 2 years. Factors that participants considered to be non-problematic included social drinking, maintaining control of their drinking, having a positive self-image, feeling that they were prepared for the effects of surgery, seeking emotional and instrumental support from people they are close to, and feelings of self-confidence that they can keep their alcohol use under control

Smith *et al*. [9] found that major depression was associated with an increased risk of AUD. In a review of studies examining alcohol and RYGB surgery, Briegleb and Hanak [11] found that male sex, younger age, regular cigarette consumption, regular alcohol consumption, pre-bariatric surgery diagnosis of AUD, recreational use of drugs, poor sense of belonging (feeling alienated), and having a RYGB procedure were all risk factors for post-surgical AUD. Bramming *et al*. [10] examined gender differences in post-bariatric surgery AUD risk and found that both men and women had an increased risk of developing AUD compared to individuals who did not have bariatric surgery. Bramming *et al*. [10] also found that age was also a risk factor, with individuals between 18 and 34 years old having the highest risk; the risk reduced with increasing age. Furthermore, Bramming *et al*. [10] found an overall increased long-term risk of AUD after bariatric surgery. Those who had bariatric surgery, mainly gastric bypass, were found to have a higher risk for AUD compared to those who did not have surgery. Mahmud *et al*. [12] found that patients receiving an RYGB had an increased risk of hospitalization for AUD.

## Discussion

The purpose of our study was to highlight the high-risk criteria mentioned in the guidelines of the American Society for Metabolic and Bariatric Surgery [19]. These criteria include a history of substance use reaction, daily pre-surgery alcohol consumption, undergoing RYGB, and smoking, which corresponds with our findings. Finding the actual prevalence of alcohol abuse during the postoperative period has also been a focus. Compared with the studies examined in this review, varying incidence rates have been indicated in the literature with evidence showing that around 3.0% of individuals undergoing surgery may experience alcohol-related abuse; reported postoperative alcohol consumption incidences ranged from 4.9% to 6.5% [7, 20].

### Risk Factors

Various risk factors contribute to alcohol problems after gastric bypass surgery, which include regular use of alcohol pre-surgery, gender, age, and the nature of surgery performed. Even though male sex was found to be a risk factor for developing AUD among bariatric surgery patients, most patients seeking inpatient treatment for AUD after bariatric surgery are female. Research by Spadola *et al*. [21] indicated that bariatric surgery patients might be over-represented in substance abuse treatment facilities; however, the authors concluded that these patients had a low prevalence of problematic alcohol use. They suggest that this finding should not prevent those needing the procedure from getting it.

Prior studies have shown that patients undergoing RYGB, one of the most common bariatric procedures, are at a much higher risk for post-surgery alcohol-related issues compared to other surgeries and pre-surgical conditions [22, 23, 24]. RYGB has been associated with a heightened risk of incident AUD symptoms within five years post-surgery [23]. A review by Gregorio *et al*. [20] found that post-operative AUD increased in their patients between 6 months and 3 years after surgery. Gregorio *et al*. [20] also showed that being male, consuming alcohol pre-surgery, and having an RYGB were also risk factors. Another review by Spadola *et al*. [21] followed a sample longitudinally and found that patients who had gastric bypass were at an elevated risk for post-bariatric surgery alcohol-use problems. Spadola *et al*. [21] also reported that the risk factors for problematic post-operative alcohol included pre-bariatric surgery problematic alcohol use, male sex, younger age, tobacco use, and symptoms of attention deficient and hyperactivity disorder.

### Psychological Factors

Our findings underscore the psychological factors at play in the development of AUD. Many individuals who undergo bariatric surgery may already have a history of disordered eating—alcohol may be a substitute behavior in the absence of food. Post-surgical psychological adjustments post-surgery, combined with the altered metabolism, can make certain individuals more vulnerable to AUD. Additionally, the studies reviewed in this paper highlight the need for preoperative counseling and postoperative monitoring. Many patients are not adequately warned about the risks of alcohol use after surgery, and ongoing education, psychological support, and monitoring for signs of AUD are crucial in preventing the development of alcohol-related problems.

### Physiological Changes in the Gut

The physiological changes induced by RYGB and SG, such as rapid gastric emptying and altered pharmacokinetics, may contribute to higher vulnerability to AUD. Despite various hypotheses into the cause of this vulnerability, including changes in neurohormonal factors and brain processing, the concept of “addiction transfer” or “symptom substitution” remains prevalent among practitioners and patients. However, empirical evidence supporting this knowledge is lacking. Some individuals may opt for lower-calorie food and alcoholic beverages, or uncarbonated options like hard beverages, which could potentially lead to higher intoxication levels due to metabolic changes. This pattern of consumption is concerning given the propensity for elevated blood alcohol concentration levels relative to the amount of alcohol consumed, especially among individuals who have undergone bariatric surgery.

Beyond metabolic changes, neurohormonal and brain processing factors likely contribute to increased alcohol sensitivity and AUD risk post-bariatric surgery. Altered gut hormones, such as ghrelin and glucagon-like peptide-1 (GLP-1), impact the brain’s reward system, influencing the brain’s reward pathways by altering dopamine signaling in the mesolimbic system, and impacting cravings and reward sensitivity. Dysregulation of these hormones in AUD may reinforce alcohol-seeking behavior through altered reward responses [25, 26]. Increased GLP-1 levels may also enhance alcohol’s effects by influencing the dopamine pathways linked to addiction [27].

AUD has been linked to alterations in the gut microbiome, which may disrupt normal brain-gut communication. These changes in gut bacteria can influence the production of neurotransmitters and inflammatory markers that interact with the central nervous system, potentially exacerbating cravings and mood disturbances associated with alcohol dependence [28].

“Addiction transfer” theory is a potential explanation for why the incidence of postsurgical AUD has risen. Although there are almost no empirical studies of addiction transfer itself [29], it has nonetheless gained the interest of researchers looking for a possible explanation for postsurgical AUD. The theory hypothesizes that neurohormonal and brain changes can heighten vulnerability to AUD by affecting reward processing and decision-making. One study has suggested that the root cause may be due to the presence of the Dopamine Receptor D2 (*DRD2*) *Taq A1 allele*, which has been found in a majority of patients with postsurgical AUD [30]. This has been dubbed “reward deficiency syndrome” and suggests that patients use food to protect against substances like alcohol; however, patients return to alcohol after surgery due to the physical changes that limit food intake. Additionally, rapid post-surgical alcohol absorption results in higher blood alcohol levels, which in turn intensifies intoxication and reinforces its use [29].

### Changes in Alcohol Consumption After Surgery

Alcohol consumption after weight loss surgery has been shown to change over time. Prior studies revealed that alcohol consumption tends to increase by about 2% each year after surgery [7, 20]. Researchers also found that the timing of alcohol intake use can vary after surgery. People are apt to drink alcohol at a lower rate and use substances less in the first 6 months after surgery. This decreased consumption may be due to doctors advising against drinking alcohol in the first 6 months after surgery. A year after surgery, this consumption can dramatically increase. Some studies found that people drank more alcohol post-surgery in about 33% of cases, while they drank less in about 13% of cases [7, 31, 32, 33, 34, 35, 36, 37, 38]. One long-term study showed that alcohol consumption decreased by about 9.1% after weight loss surgery [39].

Among individuals who underwent RYGB, there was a significant decrease in the number of drinks used on an average drinking day during the initial year after surgery [7]. There was a significant increase in the prevalence of AUD during the second postoperative year compared to the first postoperative year. Conversely, among those who received LAGB, there were no significant changes observed in either the number of drinks consumed on a typical drinking day or the prevalence of AUD over time.

### Complications

The articles reviewed highlight several important findings, particularly concerning long-term physical, psychological, and weight-related complications. One significant long-term physical complication is an increased risk of liver disease, such as alcoholic hepatitis or cirrhosis, which can develop due to chronic alcohol misuse in patients with altered alcohol metabolism following surgery. Additionally, AUD can lead to nutritional deficiencies that are already a concern post-bariatric surgery. Excessive alcohol intake can impair nutrient absorption, exacerbating deficiencies in vitamins such as B12, iron, and calcium. These deficiencies could potentially lead to anemia, osteoporosis, or neurological issues, respectively. Furthermore, there is an increased risk of weight regain, as AUD can disrupt the patient’s ability to adhere to healthy lifestyle changes, reducing the overall success of the surgery.

There are several long-term mental health complications of post-surgical AUD. Patients may experience higher rates of depression, anxiety, and social isolation, all of which can interfere with overall quality of life, possibly interfere recovery, leading to worsening post-surgery outcomes.

### Pre-Surgical Education and Post-Surgical Monitoring

While bariatric surgery can effectively manage obesity, the overall long-term complications associated with AUD—liver disease, nutritional deficiencies, mental health issues, and weight regain—highlight the need for comprehensive preoperative education and long-term postoperative monitoring. Long-term AUD is associated with a progressive accumulation of health risks that significantly impact an individual’s physical and mental well-being. Chronic alcohol consumption often leads to nutritional deficiencies, such as thiamine deficiency, which can result in severe neurological conditions like Wernicke-Korsakoff syndrome. Over time, the combination of nutritional deficits and the direct neurotoxic effects of alcohol can cause cognitive decline, memory loss, and an increased risk of dementia. Mental health issues, including depression and anxiety, also tend to worsen as social isolation, and deteriorating health reinforce psychological distress. Together, these cumulative effects of prolonged AUD contribute to a cycle that intensifies the risk and severity of both physical and mental health complications over time. Addressing these risks proactively is critical to ensuring the overall well-being of patients undergoing bariatric surgery in addition to a deeper analysis of how various factors interact over time to contribute to long-term AUD risk. Physiological shifts combined with behavioral and other psychological factors, such as preexisting addiction tendencies, emotional regulation difficulties, and social influences, can heighten the risk of developing AUD. Patients who turn to alcohol as a replacement for food are particularly vulnerable as their altered metabolism leads to quicker intoxication. Additionally, psychological stressors like depression and anxiety, which can be common after surgery, can further increase the likelihood of AUD. Without adequate postoperative support, these factors create a persistent and complex risk landscape. However, by actively monitoring these interactions, clinicians can identify at-risk patients better and implement long-term interventions to prevent the development of AUD.

### Interpretation

Overall, bariatric surgery—particularly RYGB—is associated with an increased risk of developing AUD postoperatively. This risk appears to be influenced by altered alcohol metabolism that occurs after the surgery, in which alcohol is absorbed more rapidly and reaches higher blood alcohol levels than before surgery. Consequently, patients experience stronger and more prolonged effects of alcohol, which can contribute to the development of AUD.

### Key Implications

Key implications of this study include the following:

1. **Metabolic Changes and Increased Vulnerability**: Bariatric surgery, particularly procedures like Roux-en-Y gastric bypass (RYGB), alters alcohol metabolism. Patients absorb alcohol faster, leading to higher blood alcohol concentrations and prolonged intoxication. Faster absorption can heighten the risk of developing AUD postoperatively. Clinicians must be aware of this vulnerability and monitor alcohol consumption closely in bariatric patients.

2. **Long-Term Health Complications**: Patients with AUD after bariatric surgery are at greater risk for serious complications, such as liver disease, nutritional deficiencies, and gastrointestinal issues. Alcohol misuse can impair the absorption of essential nutrients, exacerbating postoperative complications like anemia, osteoporosis, and neurological issues.

3. **Psychological and Behavioral Risks**: There is a significant psychological component since many patients use food to cope with emotional distress prior to surgery. When food is no longer an effective coping mechanism post-surgery due to physical changes, some patients may turn to alcohol, leading to addiction transfer. This shift from food to alcohol increases the risk of developing AUD.

4. **Impact on Surgery Outcomes**: Alcohol misuse post-surgery can undermine the success of the bariatric procedure. It can lead to weight regain, poorer long-term weight management, and a reduction in overall health improvement. These outcomes highlight the importance of ongoing support and monitoring for bariatric patients who are at risk of or develop AUD.

5. **Need for Comprehensive Pre- and Post-Surgery Care**: The risk of AUD underscores the need for a holistic approach to bariatric surgery that includes preoperative education about the risks of alcohol use and long-term follow-up care. Regular screening for alcohol use and mental health issues, along with access to counseling or support groups, can help prevent or mitigate the development of AUD.

### Limitations

There were several limitations to this study. First, the biggest limitation was our limited access to articles, which excluded many potential studies. Since our search resulted in several older studies, this review lacks timeliness. Second, although we reported quantitative values, this narrative review primarily presented qualitative data about each article. We did not use statistical analysis, a systematic review methodology to find articles, nor a meta-analysis methodology to synthesize data across articles. We could only describe the articles and themes found within the articles. Our results are only a summary of some of the literature available.

## Conclusions

The findings of this review highlight the significant risk of AUD in patients following bariatric surgery, particularly in those who undergo sleeve gastrectomy or Roux-en-Y gastric bypass. These results highlight the significance of thorough preoperative evaluations and continued observation of alcohol consumption patterns following surgery. It is essential to understand how altered gastrointestinal physiology, nutritional deficiencies, and AUD interact to minimize adverse outcomes and optimize postoperative care for this susceptible patient population. While the current literature provides important insights, future research should focus on further understanding the underlying mechanisms driving increased alcohol sensitivity post-surgery, including the role of neurohormonal changes and altered gut-brain signaling.

Further research is needed to elucidate the underlying mechanisms driving increased alcohol sensitivity and incidence of AUD symptoms post-surgery, which will ultimately aid in the development of effective management strategies tailored to the unique needs of patients. Specific research questions should include the following: (1) how do changes in GLP-1 and ghrelin levels influence alcohol metabolism and craving in post-bariatric patients; (2) what are the neurobiological pathways that contribute to addiction transfer from food to alcohol; (3) how effective are preoperative counseling programs in reducing the risk of AUD within the first five years after bariatric surgery; (4) what specific counseling approaches (e.g., cognitive-behavioral therapy, motivational interviewing) are most successful in mitigating the risk of developing AUD post-surgery; (5) does the timing and duration of preoperative counseling impact its effectiveness in preventing AUD in bariatric patients; (6) what role does patient education on alcohol sensitivity and addiction risk play in reducing postoperative AUD incidence; and (7) how do preoperative counseling programs influence long-term adherence to healthy coping mechanisms, and does this reduce the likelihood of addiction transfer from food to alcohol? Future research should also explore targeted interventions, such as preoperative counseling programs and long-term postoperative support systems, to mitigate the development of AUD in this population. Studies investigating the efficacy of behavioral interventions and pharmacological treatments tailored to bariatric patients with AUD would also be valuable. By addressing these questions, future research can provide a clearer understanding of AUD in bariatric surgery patients and develop effective strategies for prevention and treatment.

## Availability of Data and Materials

Not applicable.
